# Prediction of hospitalization using artificial intelligence for urgent patients in the emergency department

**DOI:** 10.1038/s41598-021-98961-2

**Published:** 2021-09-30

**Authors:** Jung-Ting Lee, Chih-Chia Hsieh, Chih-Hao Lin, Yu-Jen Lin, Chung-Yao Kao

**Affiliations:** 1 Si-Wan College, National Sun Yat-Sen University, Kaohsiung, Taiwan; 2 Department of Emergency Medicine, National Cheng Kung University Hospital, College of Medicine, National Cheng Kung University, No.138, Shengli Rd., North District, Tainan, 70403 Taiwan; 3 Department of Electrical Engineering, National Sun Yat-Sen University, Kaohsiung, Taiwan

**Keywords:** Health care, Signs and symptoms

## Abstract

Timely assessment to accurately prioritize patients is crucial for emergency department (ED) management. Urgent (i.e., level-3, on a 5-level emergency severity index system) patients have become a challenge since under-triage and over-triage often occur. This study was aimed to develop a computational model by artificial intelligence (AI) methodologies to accurately predict urgent patient outcomes using data that are readily available in most ED triage systems. We retrospectively collected data from the ED of a tertiary teaching hospital between January 1, 2015 and December 31, 2019. Eleven variables were used for data analysis and prediction model building, including 1 response, 2 demographic, and 8 clinical variables. A model to predict hospital admission was developed using neural networks and machine learning methodologies. A total of 282,971 samples of urgent (level-3) visits were included in the analysis. Our model achieved a validation area under the curve (AUC) of 0.8004 (95% CI 0.7963–0.8045). The optimal cutoff value identified by Youden's index for determining hospital admission was 0.5517. Using this cutoff value, the sensitivity was 0.6721 (95% CI 0.6624–0.6818), and the specificity was 0.7814 (95% CI 0.7777–0.7851), with a positive predictive value of 0.3660 (95% CI 0.3586–0.3733) and a negative predictive value of 0.9270 (95% CI 0.9244–0.9295). Subgroup analysis revealed that this model performed better in the nontraumatic adult subgroup and achieved a validation AUC of 0.8166 (95% CI 0.8199–0.8212). Our AI model accurately assessed the need for hospitalization for urgent patients, which constituted nearly 70% of ED visits. This model demonstrates the potential for streamlining ED operations using a very limited number of variables that are readily available in most ED triage systems. Subgroup analysis is an important topic for future investigation.

## Introduction

Visits to the emergency department (ED) have continued to increase in recent decades^1^. When the capacity of EDs cannot satisfy the demand from patients, it results in ED crowding. ED crowding has become a worldwide problem, which could significantly delay the delivery of medical care and worsen patient outcomes^2–4^.

Timely assessment to accurately prioritize patients is crucial for ED management. The triage system, along with Taiwan triage and acuity scale (TTAS), has been adopted in all EDs in Taiwan. The TTAS is a five-level system, with the most acute patients designated level-1 and the least acute as level-5^5^. The five levels of triage are therefore defined as level-1, critical; level-2, emergent; level-3, urgent; level-4, less urgent; and level-5, non-urgent. Among ED patients, approximately 25% are critical or emergent. The majority of ED patients were triaged as urgent, comprising up to 70% of patients^6^. More than 20% of urgent patients, after primary management in the ED, need admission^1^. The large number and complicated composition of urgent patients have become a challenge since both under-triage (i.e., “critical” or “emergent” patients being triaged as “urgent”) and over-triage (i.e., “less urgent” or “non-urgent” patients being triaged as “urgent”) often occur^7,8^. Moreover, the accuracy of triage is easily affected by the operator’s experience and subjective judgment^9^.

Electronic triage using artificial intelligence (AI) has been proposed to predict patient outcome^10^. The aim of this study was to develop a computational model using AI methodologies to accurately predict patient outcomes—i.e. whether the patient requires hospitalization—using data that are readily available in most ED triage systems. The target group in this study was urgent (level-3) patients since this group of patients constitutes the majority of ED visits and encounters the greatest challenge with respect to under-triage and over-triage. With the assistance of this AI model, the need of hospitalization for urgent patients could be more accurately assessed, which in turn could optimize risk stratification and streamline management of patients.

## Methods

### Study design and setting

This study retrospectively collected data from the ED of a tertiary teaching hospital between January 1, 2015, and December 31, 2019. The study hospital, located in Tainan city, Taiwan, has 914 general ward beds and 214 intensive care beds. The average number of ED visits is 100,668 annually. A computerized system, the so-called Taiwan triage and acuity scale (TTAS) similar to ESI, was adopted in the ED for triage. The study only included urgent (level-3) triaged visits with complete information at triage and recorded disposition of either admission or discharge. Individuals with any missing information at triage and other dispositions, such as transfer or against-advice discharge, were excluded. The primary outcome was hospital admission.

### Data collection and processing

In total, eleven variables were used for data analysis and prediction model building. These include one response variable, two demographic variables, and eight clinical variables. The clinical variables included six vital signs used for triage evaluation, medical history, and chief complaints. Among the eleven variables, the response and gender variables are binary, while the other variables are numeric in nature.

### Response variable

The primary response variable was the patients’ disposition made by ED physicians and was encoded as a binary variable with 'admission' coded as 1 and 'discharge' coded as 0.

### Demographics

Two demographic variables, age and sex, were used in this study. They were either collected from the patients or from the Taiwan Health Care Database System by the triage nurse. While the age variable is numeric with one decimal fraction, and the gender variable is binary with the 'male' gender coded as 1 and the 'female' gender coded as 0.

### Vital signs for triage evaluation

Six vital signs, including temperature, heart rate, respiratory rate, systolic blood pressure, diastolic blood pressure, and mean arterial pressure (MAP), were measured and recorded by the triage nurse. Oxygen saturation was not included, as a significant number of patients did not have their oxygen saturation level measured at triage. As such, the amount of missing information made this vital sign unavailable for model building. All six selected variables are numeric. Except for the temperature variable, which has one decimal fraction, the other variables have integer values.

### Medical history

Electronic medical records of the patients were pulled out by the triage nurse while patients arrived at the triage station using the International Classification of Diseases (ICD) code. Diseases were classified following the ICD-10 codes, and an integer score was assigned to each classification. The value of this variable is the sum of scores of all classifications the patient belongs to or zero if the patient had no medical history available. The value of this variable ranges from 0 to 12.

### Chief complaints

The Taiwan triage acuity scale is defined by the Ministry of Health and Welfare (MOHW) of Taiwan. It includes a code system corresponding to the patient’s chief complaint and its severity, which is similar to the ICD code. There are four major categories in this code system: trauma, nontraumatic adult, pediatrics, and environmental emergency. The triage nurse chooses the code that meets the patient’s chief complaint and its severity most appropriately and then decides the patient’s emergency severity index.

$$v\left({\text{cf}}\right)=\frac{{n}_{h,r}(\text{cf)+}{n}_{h,d}(\text{cf)}}{{n}_{t}(\text{cf)}}+\frac{{n}_{h,d}(\text{cf)}}{{n}_{h,r}(\text{cf)+}{n}_{h,d}(\text{cf)}}$$

where

- $v\left({\text{cf}}\right)$: decimal value for the given chief complaint code cf.
- ${n}_{t}(\text{cf)}$: the number of patients in the training data set whose chief complaint code is cf.
- ${n}_{h,r}(\text{cf)}$: the number of patients in the training data set whose chief complaint code is cf and who were hospitalized and eventually recovered.
- ${n}_{h,d}(\text{cf)}$: the number of patients in the training data set whose chief complaint code is cf and who were hospitalized and eventually deceased.

Mathematically speaking, the value $v\left({\text{cf}}\right)$, being the sum of two terms, represents the risk of hospitalization for patients with chief complaint code cf. The first term is the percentage of patient hospitalizations with chief complaint code cf, while the second term is the percentage of deaths among patients with chief complaint code cf who were hospitalized. The second term is introduced to differentiate one chief complaint from another, which results in the same number of admitted patients. Intuitively, a medical complaint that results in more deceased patients should be assigned a higher risk value. Note that when ${n}_{t}(\text{cf)}$ is equal to zero, which means that there is no patient in the training data set whose chief complaint code is cf, $v\left({\text{cf}}\right)$ is set to zero.

### Model development, fitting, and evaluation

A prediction model for hospital admission was developed using neural network and machine learning methodologies^11^. A three-layer structure is assumed with output dimensions of 100, 12, and 1. Between layers, the batch normalization technique was adopted to facilitate the training process. The (final) output layer utilizes the sigmoid function as the activation function. As such, a mathematical representation of the model can be expressed as

$$y=\frac{1}{1+{e}^{f(x)}}$$

where $x$ represents the input vector of 10 variables, and $y$ is the model output, which has a value between 0 and 1. The model output represents the likelihood of the patient being admitted. The nonlinear function $f(x)$, primarily determined by the first two layers, contains 2549 trainable model parameters.

The data were randomly divided into training (80%) and validation (20%) sets. Statistical similarity of the training and validation sets regarding demographics, medical history, and chief complaints was confirmed by statistical analysis. Specifically, the percentages of admitted and discharged patients in each category of the training and validation data sets were calculated and compared. The model parameters were trained based on the training data set, and the predictive power of the model was evaluated by the validation data set using the area under the curve (AUC) in the receiver operating characteristic (ROC) analysis. The optimal cutoff point on the ROC curve was calculated based on Youden's index^12^, which in turn was used to calculate sensitivity, specificity, positive predictive value, and negative predictive value for the prediction model applied to the validation data.

### Testing the benefit of additional training samples

To answer a key question of whether one can improve the performance of the model if additional training samples are used for tuning the model parameters, we trained the model on randomly selected fractions of the training set. Specifically, we trained the model using 100%, 75%, 50%, 25%, and 12.5% of the training samples, calculated the corresponding AUCs on the held-out validation set and quantified the incremental gain in performance.

### Variables of importance

To determine which variables are crucial and more "useful" for predicting hospital admission, lower dimension models were trained using various subsets of variables. The performances of these models, indicated by AUCs on the validation set, were compared to determine whether these models could predict hospital admission as robustly as the full model. Variables to drop were selected based on the statistical analysis shown in Tables 1 and 2 and physician experiences.

**Table 1:** Descriptive analysis of all patients included in the training and validation sets.

	Training set (80%)				Validation set (20%)			
	Admitted patients (n = 35,831, 15.75%)	Discharged patients (n = 191,696, 84.25%)	Total patients (n = 227,527)	p value	Admitted patients (n = 8991, 15.81%)	Discharged patients (n = 47,889, 84.19%)	Total patients (n = 56,880)	p value
**Gender**				< 0.001				< 0.001
Male	18,979 (52.97%)	90,996 (47.47%)	109,975 (48.33%)		4719 (52.49%)	22,739 (47.48%)	27,458 (48.27%)	
Female	16,852 (47.03%)	100,700 (52.53%)	117,552 (51.67%)		4272 (47.51%)	25,150 (52.52%)	29,422 (51.73%)	
**Age**				< 0.001				< 0.001
0–17	5005 (13.97%)	43,789 (22.84%)	48,794 (21.45%)		1300 (14.46%)	10,890 (22.74%)	12,190 (21.43%)	
18–64	15,298 (42.69%)	110,165 (57.47%)	125,463 (55.14%)		3870 (43.04%)	27,442 (57.30%)	31,312 (55.05%)	
65–84	12,074 (33.70%)	31,224 (16.29%)	43,298 (19.03%)		2918 (32.45%)	7911 (16.52%)	10,829 (19.04%)	
≥85	3454 (9.64%)	6518 (3.40%)	9972 (4.38%)		903 (10.04%)	1646 (3.44%)	2549 (4.48%)	
**Medical history**				< 0.001				< 0.001
With	16,844 (47.01%)	28,838 (15.04%)	45,682 (20.08%)		4149 (46.15%)	7309 (15.26%)	11,458 (20.14%)	
Without	18,987 (52.99%)	162,858 (84.96%)	181,845 (79.92%)		4842 (53.85%)	40,580 (84.74%)	45,422 (79.86%)	
**CC category**				< 0.001				< 0.001
Nontraumatic adult	27,706 (77.32%)	123,662 (64.51%)	151,368 (66.53%)		6939 (77.18%)	30,904 (64.53%)	37,843 (66.53%)	
Pediatrics	4747 (13.25%)	38,998 (20.34%)	43,745 (19.23%)		1239 (13.78%)	9697 (20.25%)	10,936 (19.23%)	
Trauma	3343 (9.33%)	28,188 (14.70%)	31,531 (13.86%)		802 (8.92%)	7079 (14.78%)	7881 (13.86%)	
Env. emergency	35 (0.10%)	848 (0.44%)	883 (0.39%)		11 (0.12%)	209 (0.44%)	220 (0.39%)	

*CC* chief complaint, *Env. emergency* environmental emergency.

**Table 2:** Characteristics concerning age, triage evaluation, and risk value of chief complaint for all study samples.

	Discharged (84.24%) (mean, 95% CIs)	Admitted (15.76%) (mean, 95% CIs)	p value
Age	39.5 (39.39–39.59)	55.05 (54.8–55.29)	< 0.001
Temperature	37.04 (37.04–37.04)	37.24 (37.23–37.25)	< 0.001
Heart rate	96.74 (96.64–96.84)	98.39 (98.18–98.6)	< 0.001
Respiratory rate	19.86 (19.84–19.87)	19.73 (19.7–19.76)	< 0.001
Systolic blood pressure	133.72 (133.62–133.82)	135.32 (135.09–135.56)	< 0.001
Diastolic blood pressure	82.59 (82.53–82.66)	80.63 (80.48–80.78)	< 0.001
Mean arterial pressure	99.30 (99.23–99.37)	98.53 (98.37–98.69)	< 0.001
Medical history score	0.2619 (0.2554–0.2685)	0.8781 (0.8735–0.8826)	< 0.001
Risk value of chief complaint	0.1403 (0.1399–0.1407)	0.2494 (0.2478–0.2509)	< 0.001

*CIs* confidence intervals.

### Ethical approval

The study was approved by the Institutional Review Board of the National Cheng Kung University Hospital, Tainan, Taiwan (A-ER-108-451).

## Results

### Characteristics of study samples

There were 441,782 ED visits between January 2015 and December 2019. Approximately 70% of visits were classified as level-3 by the triage nurse. After the exclusion of any missing information at triage stations and other dispositions, such as transfer and against-advice discharge, a total of 282,971 samples of level 3 visits were used for this study. The visits represented 180,603 unique patients, for an average of 1.57 visits per patient. The characteristics of the study samples are shown in Tables 1 and 2.

### Model performance

The model we presented shows good distinguishing power for predicting hospital admissions using only a few metrics collected at triage stations. This model achieved a validation AUC of 0.8004 (95% CI 0.7963–0.8045). The optimal cutoff value found by Youden's index for determining hospital admission was 0.5517. Using this cutoff value, the sensitivity was 0.6721 (95% CI 0.6624–0.6818), and the specificity was 0.7814 (95% CI 0.7777–0.7851). The positive predictive value (PPV) was 0.3660 (95% CI 0.3586–0.3733), and the negative predictive value (NPV) was 0.9270 (95% CI 0.9244–0.9295).

**Figure 1:**
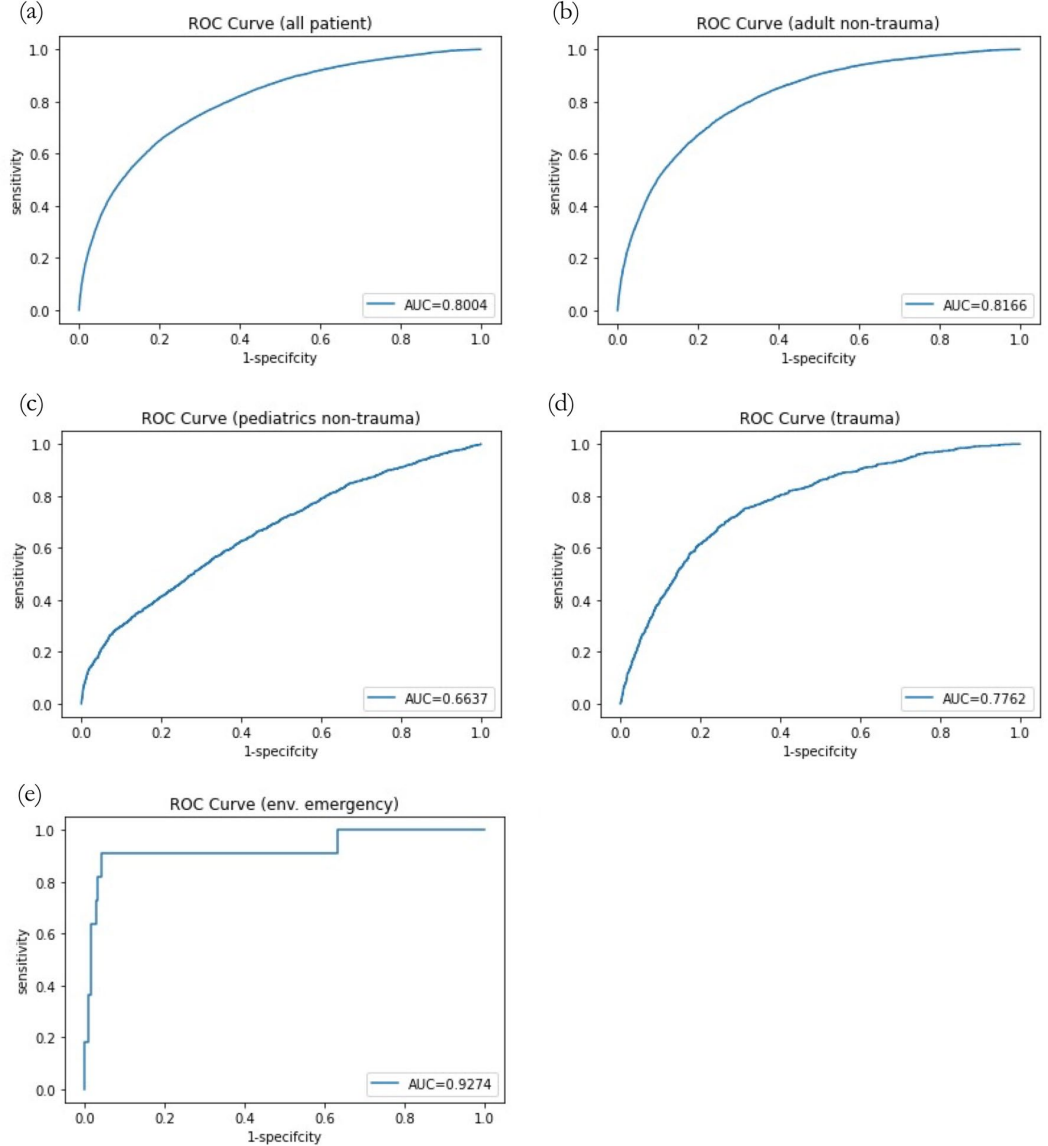
The receiver operating characteristic (ROC) curves of the predictive model for all patients and subgroups. (**a**) All patients. (**b**) Nontraumatic adult patients. (**c**) Pediatric patients. (**d**) Traumatic patients. (**e**) Patients of environmental emergency.

The model was also applied to patients in the following four subgroups to examine its respective predictive power in these groups: nontraumatic adult, pediatrics, trauma, and environmental emergency. For nontraumatic adult patients, the model achieved a validation AUC of 0.8166 (95% CI 0.8199–0.8212), which was higher than its performance on all patients. For pediatric and traumatic patients, however, the model performances were worse. The achieved validation AUCs were 0.6637 (95% CI 0.6492–0.6782) and 0.7762 (95% CI 0.7623–0.7901), respectively. For patients with environmental emergencies, the model performed significantly higher, with a validation AUC of 0.9274 (95% CI 0.8801–0.9747). All validation ROC curves are shown in Fig. 1.

### Testing the benefit of additional training samples

The model trained on 75% of the training set achieved a validation AUC of 0.7999 (95% CI 0.7958–0.8040). The 95% confidence interval contains the AUC of the model trained on the entire training set. The algorithm with the proposed model structure appears to reach maximum performance at 75% of the training set or less. All AUC values with corresponding 95% confidence intervals are provided in Table 3.

**Table 3:** Validation area under the curve (AUC) values and the corresponding 95% confidence intervals of models trained on various percentages of the training data set samples.

Percentage of training set samples (%)	AUC (95% confidence intervals)
100	0.8004 (0.7963, 0.8045)
75	0.7999 (0.7958, 0.8040)
50	0.7956 (0.7914, 0.7998)
25	0.7849 (0.7806, 0.7892)
12.5	0.7558 (0.7511, 0.7647)

### Variables of importance

Excluding four vital signs (temperature, respiratory rate, systolic blood pressure, and diastolic blood pressure), a lower-dimensional model was built using the remaining six variables (age, sex, heart rate, MAP, medical history, and chief complaint). The model achieved a validation AUC of 0.7963 (95% CI 0.7921–0.8005); the 95% CI contains the AUC of the full-dimensional model. Notably, if one further excludes the chief complaint variable, the predictive power of the resulting model diminishes significantly. The validation AUC of the model built on only five variables (age, sex, heart rate, MAP, and medical history) dropped to 0.7501 (95% CI 0.7454–0.7548). To further confirm this observation, a model was built based on nine variables (all but the chief complaint variable). The validation AUC of this model also dropped to 0.7517 (95% CI 0.7470–0.7564).

## Discussion

After emergency physicians examine patients for the first time, they often make predictions regarding the patients’ outcomes and diagnoses using so-called diagnostic intuition^13^. Physicians often work using two types of mindsets: the intuition mindset and the analytical mindset^14^. The intuition mindset depends largely on the physicians’ own experiences. It responds quickly and works similar to pattern recognition. The analytical mindset is usually based on the existing knowledge and available data. These processes are complicated and cognitively resource-demanding^15^. The intuition mindset plays an important role in medical decision making^16^. It is often associated with the patient’s prognoses rather than diagnoses^17^. The work based on this mindset benefits from the physician’s clinical reasoning and the accuracy of the diagnoses^18^. The setting of our study simulates the scenario where the physician visits the patient for the first time. The computational model predicts the patient’s outcome with very limited information; the work of this model is comparable to the physician’s diagnostic intuition. The existing clinical decision-support systems are often criticized for their nonuser friendly methods of information collection^19^. Our model avoids these drawbacks by adopting variables that can all be extracted from the electronic medical record system automatically. This is a significant advantage for the utilization of our model.

The primary purpose of this model was to predict whether an urgent (level-3) patient requires hospitalization right after they are initially triaged. The model was designed to serve as a secondary triage tool assessing probability of hospitalization, which essentially gives an indication of the severity level of the patient. The assessment that our model provides would help the risk stratification of patients and streamlining the ED operations^10^. Patients who were predicted requiring hospitalization would be sent to the therapeutic area for timely examination and further treatment or who otherwise could be fast-tracked for rapid evaluations and discharge. This in turn improves both the quality of medical care and patient safety^20^. By reducing unnecessary examinations and the length of ED stay, the model could also improve patient’s satisfaction^21–23^.

With the assessment provided by the model, ED physicians would be more confident in their decisions regarding patient disposition. For patients who need hospital admission, the process can be initiated earlier according to the prediction given by the model to reduce ED boarding^24,25^. On the other hand, unnecessary examination and observation in the ED could be avoided for those who do not need hospital admission. Furthermore, based on the prediction of the need for hospitalization, the ED can streamline patients to primary care services, which subsequently reduces ED crowding^26,27^. The efficient allocation of medical resources in the ED can improve the cost management and quality control^28^.

Because only a few variables are adopted in our model and they are readily available, the model can be utilized in the prehospital setting to improve the efficiency of the emergency medical services (EMS) system. With the aging of the population, the loading of the EMS has increased in many countries^29^. Misuse of the ambulance by low-acuity patients unnecessarily occupies emergency medical resources and thus endangers patients who truly need emergent medical aid^30^. Our model can be used by the EMS system to divert ambulance requests to other alternatives for those with low acuity^31^. The potential benefits include better reserve of the EMS resources and possible improvement in the outcome of patients who receive medical aid sooner^32–34^. For the patients who were predicted not requiring hospitalization, the emergency medical technicians could apply a “treat and release” protocol by giving primary medical aids in the field without transporting the patients to hospitals^35,36^.

Another potential application of our model is “self-triage”. Certain computer algorithms had been proposed for patients to perform self-triage before ED visits, in the hope of better patient streamlining^37–40^. However, most of the algorithms either require many variables to perform prediction or fail to demonstrate a sufficient prediction power^37–40^. Our model offers a reliable prediction of hospital admission using very limited variables that could be obtained by the patients themselves. Those who were predicted to have low possibilities of hospitalization could be first delivered to primary care services.

People generally believe that having many decision variables is necessary for successfully building a predictive model based on machine learning algorithms. On the other hand, having many decision variables often makes it difficult to explain the possible causality and correlation between the decision variables and the designated model output^41^. In this study, we did not blindly use many variables for the model building process. Instead, we chose specific variables that may have explainable causality with the model output according to the physicians' experiences. Moreover, important information, such as chief complaints and medical history, were distilled into one single variable representing the risk of hospitalization. This is in contrast to the approach taken in other studies^42^. The results of our study demonstrate that this approach works very well. Models with good predictive power can be built using as few as six decision variables. Compared to previous methods^42,43^, very few computational resources are required for building and using our model, and the predicted outcomes are easily explained in medicine and conform to medical intuition.

In our study, we found that the predictive power of our model differed among the four subgroups (nontraumatic adult, pediatrics, trauma, and environmental emergency) of ED patients. The model performed worse in the traumatic subgroup and substantially worse in the pediatric nontraumatic subgroup. Technically speaking, the predictive power of the model originates from recognizing certain crucial characteristics of the patients. Our results indicate that patients in one subgroup appear to have crucial characteristics that significantly differ from those of patients in another subgroup. For example, among nontraumatic adult patients, the likelihood of hospitalization increases as age grows, while among pediatric patients, this correlation is usually the opposite. In contrast, among traumatic patients, 'age' may not be an important factor for predicting hospitalization at all. As such, it seems difficult to use a single model to predict the outcome for all types of patients in the ED due to the high heterogeneity among patient characteristics in different subgroups. Further studies analyzing these patient subgroups are required to build separate models with high predictive power for different patient subgroups.

## Limitations

There are some limitations in our study. First, the model was structured based on the data of single medical center. For broader utilization, it might need the data from multiple medical centers and rural hospitals to improve the accuracy of the model. Furthermore, the oxygen saturation level was not included as a variable in our model. During the study period, the triage nurses in the study hospital were not required to routinely obtain the oxygen saturation levels. As a result, the triage staff tended to skip taking measurement of the oxygen saturation level when they were subject to heavy workload. The workflow protocol was later corrected to demand this item, but the problem of data missing in our study period remains. Moreover, our model, aimed to promptly identify patients with high possibility of hospitalization, was designed to use only the data that could be collected at the initial triage. Further studies are planned to evaluate the benefits between timely prediction and the degree of accuracy when more variables, such as results of initial laboratory test and medical images, are introduced to the model. Finally, we did not perform a comparison analysis with other computer or human (physicians or triage nurses) prediction models. To our knowledge, despite certain studies on triage accuracy^37–39^, no model for prediction of hospitalization had been reported. This is an interesting subject for future studies, which would facilitate the integration of the AI model into the work of the triage crew.

## Data Availability

All the data used to draw the conclusions of this paper are available in the data presented in the figures and/or tables. The raw/processed data required to reproduce these findings are available from the corresponding author upon request.
